# Transcatheter Aortic Valve Replacement After David Procedure for Severe Aortic Regurgitation

**DOI:** 10.1016/j.jaccas.2026.108468

**Published:** 2026-05-29

**Authors:** Ahmad Makhdoum, Muhammed Suleman, Warkaa Shamkhani, Hatim AlRaddadi, Victor Chu, Tej Sheth

**Affiliations:** a Unit of Cardiac Surgery, Department of Surgery, King Abdulaziz University, Jeddah, Saudi Arabia; b Division of Cardiology and Cardiac Surgery, McMaster University, Hamilton, Ontario, Canada; c Keele Cardiovascular Research Group, Centre for Prognosis Research, Keele, United Kingdom

**Keywords:** aortic insufficiency, David procedure, TAVR, valve sparing aortic root replacement

## Abstract

**Background:**

Transcatheter aortic valve replacement (TAVR) for aortic insufficiency (AI) after valve-sparing aortic root replacement (VSARR) presents unique anatomical and procedural challenges.

**Case Summary:**

We report successful transfemoral TAVR in a 68-year-old man with severe AI following a prior David procedure. Procedural planning incorporated gated computed tomography assessment of graft dimensions and coronary heights. Device sizing and deployment strategy relied on the left main (LM) ostium as a fluoroscopic landmark and the graft diameter to define the anchoring zone. A balloon-expandable valve was selected with >15% oversizing. The device was deployed successfully without residual regurgitation or coronary obstruction.

**Discussion:**

This case highlights the feasibility of TAVR after VSARR with AI and emphasizes the importance of integrating prior surgical technique and graft anatomy into transcatheter procedural planning.

**Take-Home Message:**

Procedural planning using anatomical landmarks may aid in the safe deployment of TAVR in failed VSARR with AI.

## Visual Summary

**Figure:**
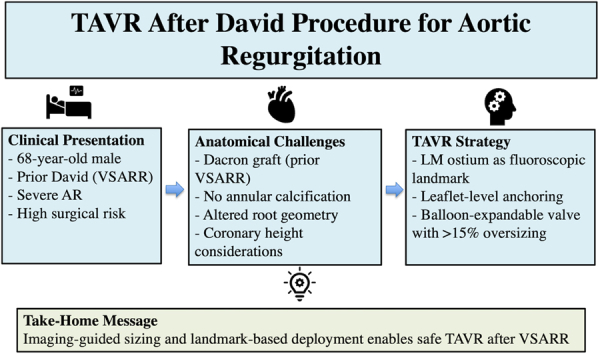

## History of Presentation

A 68-year-old man presented with a several-month history of increasing shortness of breath (NYHA functional class III symptoms) and a prior valve-sparing aortic root replacement (VSARR) (David procedure) using a 32-mm cylindrical Dacron graft and coronary artery bypass grafting with a saphenous vein graft to the right coronary artery. A transthoracic echocardiogram revealed a tricuspid aortic valve with an ejection fraction (EF) of 44%, severe aortic insufficiency (AI) due to cusp prolapse, a mean gradient of 3.7 mm Hg, and a peak gradient of 6.7 mm Hg.

**Take-Home Message**

• Procedural planning using anatomical landmarks may aid in the safe deployment of transcatheter aortic valve replacement in failed valve-sparing aortic root replacement with aortic insufficiency.

## Past Medical History

His past medical history included coronary artery disease, aortic root aneurysm, hypertension, hyperlipidemia, and prostate cancer.

## Differential Diagnosis

Congestive heart failure due to severe AI was the principal diagnosis based on echocardiogram findings. The progression of his known coronary artery disease was considered.

## Investigations

**Figure 1:**
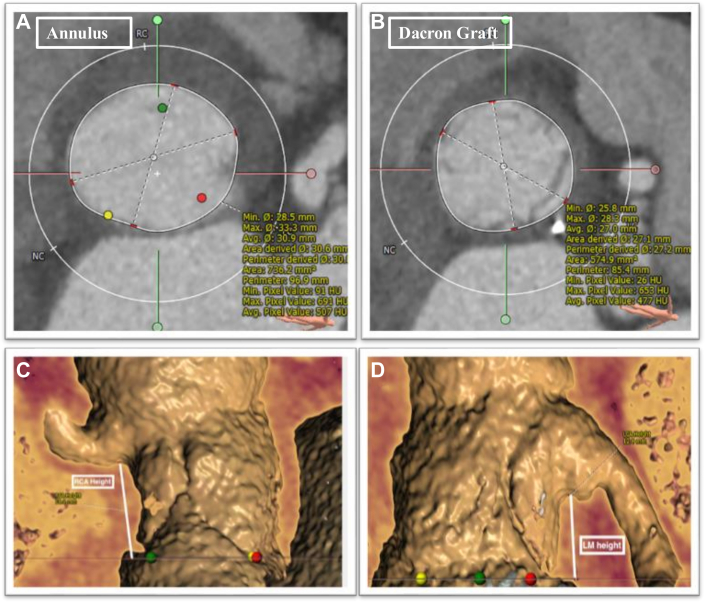
Preprocedural Gated Computed Tomography Measurements (A) Annular, (B) Dacron graft measurement, (C) right coronary artery (RCA), and (D) left main coronary artery (LM).

A multidisciplinary team approach including a cardiac surgeon, an interventional cardiologist, and a heart failure specialist was used. Because of his elevated surgical risk, declining EF, and preference to avoid reoperation, a gated computed tomography scan was performed for transcatheter aortic valve replacement (TAVR) procedural planning. Imaging (Figure 1) showed an annular area of 736 mm^2^ and a left ventricular outflow tract area of 745 mm^2^, with an area-derived diameter of 30 mm, a Dacron graft diameter of 32 mm at the left ventricular outflow tract area, and an average graft diameter of 27 mm (28.2 × 25.8 mm) at the leaflet level. The reimplanted coronary artery heights were 12.5 mm for the left main (LM) and 17 mm for the right coronary artery with a simulated valve-to-coronary distance of 5.2 mm. Preprocedural considerations focused primarily on TAVR valve size, deployment location, and maintaining adequate coronary access post-TAVR. Based on leaflet-level graft diameter and expected fibrosis, a 29-mm balloon-expandable valve (BAV) with +1 cc volume was chosen, resulting in an oversizing of >15% for sealing and anchoring stability.

## Management

**Figure 2:**
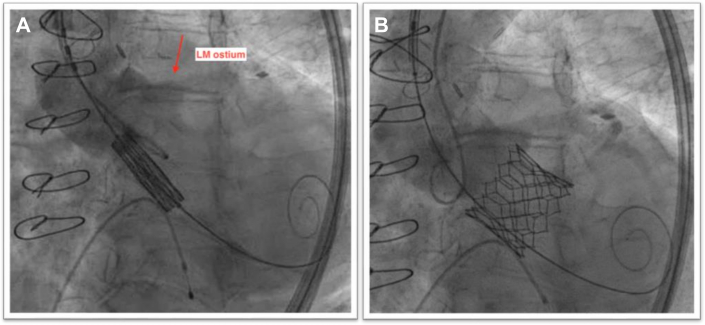
Fluoroscopic Images Demonstrating Landmark-Based Valve Positioning and Final Deployment (A) Left main (LM) ostium as a landmark for positioning and (B) final deployment.

The procedure was performed with conscious sedation. Device access was obtained with ultrasound-guided puncture and achieved through a 16-F TAVR eSheath (Edwards Lifesciences) placed in the right common femoral. Secondary access was established in the left common femoral artery with a 5-F aortic valve that was crossed with a straight stiff wire (Boston Scientific) and exchanged with a Safari stiff support wire in the left ventricle. A 29-mm balloon-expandable valve (SAPIEN 3, Edwards Lifesciences) was advanced to the root. The coplanar angle and valve positioning were carefully assessed, with the LM ostium serving as a landmark for the upper portion of the valve frame, which exhibits less foreshortening compared with the valve's lower end (Video 1, Figure 2). The device was then deployed under rapid right ventricular pacing. A total of 34 cc was delivered. After deployment, the aortic root angiogram showed mild AI. One additional cc was added for post-dilation (Video 2, Figure 2). The final angiogram showed no AI and no coronary obstruction.

## Outcome and Follow-Up

Postoperative transthoracic echocardiography showed no transvalvular or paravalvular leak, a mean gradient of 2.7 mm Hg, and a left ventricular EF of 44%. At 2-month follow-up, transthoracic echocardiography confirmed stable valve function with no AI and a left ventricular EF of 43%. The patient remained asymptomatic, and an electrocardiogram demonstrated sinus rhythm without any conduction abnormalities.

## Discussion

TAVR in AI remains a technical challenge, primarily due to the lack of annular calcification needed for predictable anchoring and sealing. In patients with prior David procedure, additional difficulties arise from the presence of noncompliant Dacron graft, resuspended commissures, and altered root geometry that may constrain the annulus and modify the effective landing zone. These anatomical factors necessitate careful planning and integration of annular measurements, leaflet-level graft dimensions, and coronary heights to guide safe device sizing and deployment.^1^^,^^2^

VSARR includes different surgical techniques with important implications for subsequent TAVR. The David procedure is a reimplantation technique in which the native annulus and root are circumferentially supported within a synthetic graft. In contrast, the Yacoub procedure is a remodeling technique that reconstructs the sinuses while preserving native annular dynamics and typically provides less subannular support. This distinction is clinically relevant, as the rigid annular support after reimplantation may facilitate anchoring of BAVs, whereas remodeling techniques may result in more compliant geometry and a higher risk of valve migration or suboptimal sealing. Variability also exists within reimplantation procedures. Straight cylindrical grafts provide uniform radial constraint but may limit sinus space and coronary clearance compared with Valsalva grafts that recreate pseudosinuses. In addition, the number and distribution of subannular sutures influence basal ring rigidity. Classical reimplantation with multiple pledgeted sutures may offer a stable anchoring platform favoring BAVs, whereas modified techniques with fewer fixation sutures may require greater device oversizing.

Valve selection is important in AI with VSARR. Traditionally, self-expandable valves are preferred for noncalcified AI because they are typically oversized by approximately 30%, compared with BEVs, which are usually oversized by 5% to 10%.^3^ However, in younger patients, balloon-expandable devices are often favored due to their shorter frame, which allows better coronary access and facilitates future reinterventions.^4^ BEVs can offer a strong radial force, which can improve sealing in noncalcified annuli or grafts. Their shorter frame and open cell design can also facilitate coronary access and repeatability, certainly an important consideration in younger patients or those with patent bypass grafts. Our case, along with those of Anzai et al^1^, ^5^ and Koren et al,^1^, ^5^ used BEV with good intraprocedural and postprocedural clinical outcomes. In contrast, both Oztas et al^6^ and Tay et al^7^ deployed a self-expanding Evolut valve. Although Evolut allows gradual deployment and recapture, which is beneficial in large annuli and uncertain anatomy, their taller frame and supra-annular leaflet position may complicate future coronary access. In addition, both cases faced critical intraprocedural difficulties with multiple deployment attempts, challenges with fluoroscopic visualization, and initial embolization. Certainly, newer and specific TAVR valves designed for AI such as the JenaValve can improve the TAVR ability in AI with its unique clipping mechanism that anchors the positioning feelers to the native valve leaflet without relying on annular calcium or the need for oversizing.^8^ However, it is in the early phases and lacking commercial availability to ascertain its efficacy in AI.

Precise deployment strategy is essential in the absence of annular calcification. In our case, the LM ostium served as a fluoroscopic landmark, and graft diameter at the leaflet level was used to define the anchoring zone. Anzai et al^1^ anchored the Sapien valve between the sinus suture line and CoreKnots, using these points as a reference within the Valsalva graft. Similarly, Tugulan et al^9^ relied on pledgeted annular sutures for anchoring, aided by fluoroscopic visualization of CoreKnots. Tay et al^7^ used a 3-dimensional transesophageal echocardiogram to overcome poor angiographic visualization in a long 8 Gelweave graft, highlighting the value of multimodal imaging in complex anatomies. These variations show the importance of adapting deployment depth and technique to each patient's specific anatomy.

Only a limited number of case reports have described TAVR after prior valve-sparing root replacement, with generally favorable procedural outcomes but variable deployment strategies, stressing the value of individualized procedural. Imaging-driven valve selection, attention to coronary anatomy, and anchoring zone were recurring themes among the reports. As TAVR use in pure AI expands, especially in cases with prior VSARR, accumulating procedural experience and long-term outcome data will be necessary to guide optimal procedural planning and practices.

**Visual Summary:**
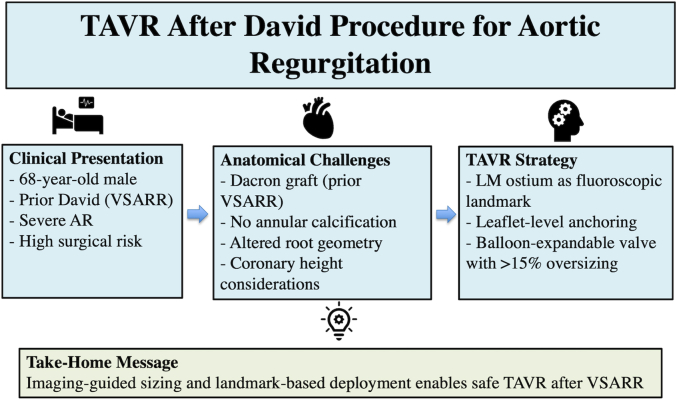
Clinical Presentation, Anatomical Challenges, and Procedural Strategy for Transcatheter Aortic Valve Replacement After the Prior David Procedure AR = aortic regurgitation; LM = left main; TAVR, transcatheter aortic valve replacement; VSARR, valve-sparing aortic root replacement.

## Conclusions

TAVR after VSARR is feasible in carefully selected patients with severe AI. This case illustrates how integrating prior surgical techniques, graft configuration, and multimodality imaging can guide device selection and deployment strategy in anatomically complex settings.

## Funding Support and Author Disclosures

The authors have reported that they have no relationships relevant to the contents of this paper to disclose.

## Appendix

Video 1 Left Main as a Fluoroscopic Landmark for Valve Positioning and Deployment.

Video 2 Final Aortic Root Angiogram After Valve Dilation.
